# Cervical Pilocytic Astrocytoma Mimicking Spondylotic Myelopathy: A Case Report and Literature Review

**DOI:** 10.7759/cureus.70296

**Published:** 2024-09-26

**Authors:** Morgane Soler-Rico, Lina Daoud, Edward Fomekong

**Affiliations:** 1 Neurosurgery, Cliniques Universitaires Saint-Luc (UCLouvain Saint-Luc), Brussels, BEL; 2 Pathology and Laboratory Medicine, Cliniques Universitaires Saint-Luc (UCLouvain Saint-Luc), Brussels, BEL

**Keywords:** myelopathy, pilocytic astrocytoma (pa), spinal cord tumor surgery, spondylotic myelopathy, tumor surgery

## Abstract

Spinal cord pilocytic astrocytoma (PA) is a rare, low-grade tumor in adults, typically presenting with a cystic component. Correct diagnosis is crucial, as gross total resection may improve survival rates. We report the case of a 44-year-old patient with chronic neck and arm pain, along with hypoesthesia on the left side, initially suspected of having myelitis based on MRI findings. Surgical exploration enabled near-complete resection, and histopathology confirmed the diagnosis of PA. This case highlights the importance of considering PA in the differential diagnosis of inflammatory and degenerative spinal cord disorders and suggests that further investigation is needed into its treatment and potential recurrence.

## Introduction

Spinal cord pilocytic astrocytoma (PA) is a World Health Organization grade 1 tumor that affects children and young adults. However, it is uncommon in adults, comprising only about 0.8% of spinal cord tumors [1]. PA are typically well-circumscribed, slow-growing lesions and often contain a cystic component [2].

The mainstay treatment for PA involves surgical intervention, and gross total resection is the goal. Achieving this goal is associated with favorable outcomes, with five-year survival rates around 84% [3].

The rarity of this condition in adults poses significant diagnostic challenges. Moreover, radiological diagnosis can be difficult, and it can mimic other spinal cord pathologies such as myelitis or other intramedullary tumors. However, accurate diagnosis is crucial to prevent delays in appropriate treatment, which can significantly influence patient outcomes.

This case report describes a patient with PA mimicking spondylotic myelopathy, illustrating the diagnostic complexities and the importance of considering PA in differential diagnoses for similar presentations.

## Case presentation

**Figure 1:**
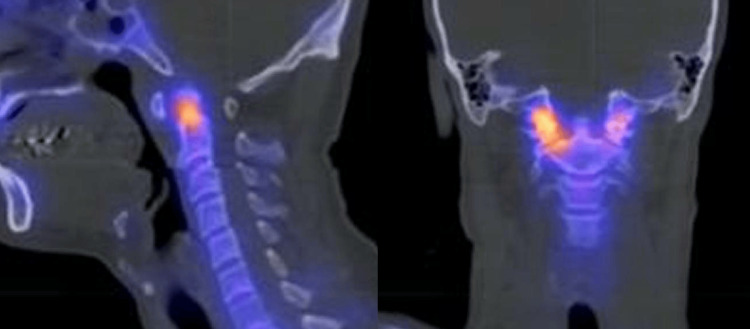
SPECT images revealed atlanto-axial arthrosis with active C1-C2 and occipital diastasis SPECT: single-photon emission computed tomography

A 44-year-old patient presented with chronic bilateral C6 and C7 cervicobrachialgia persisting for six years and left-sided hemihypoesthesia and paresthesias for one year. Imaging evaluation revealed atlanto-axial arthrosis with active C1-C2 and occipital diastasis and suspected C2-C4 myelitis (Figure 1).

**Figure 2:**
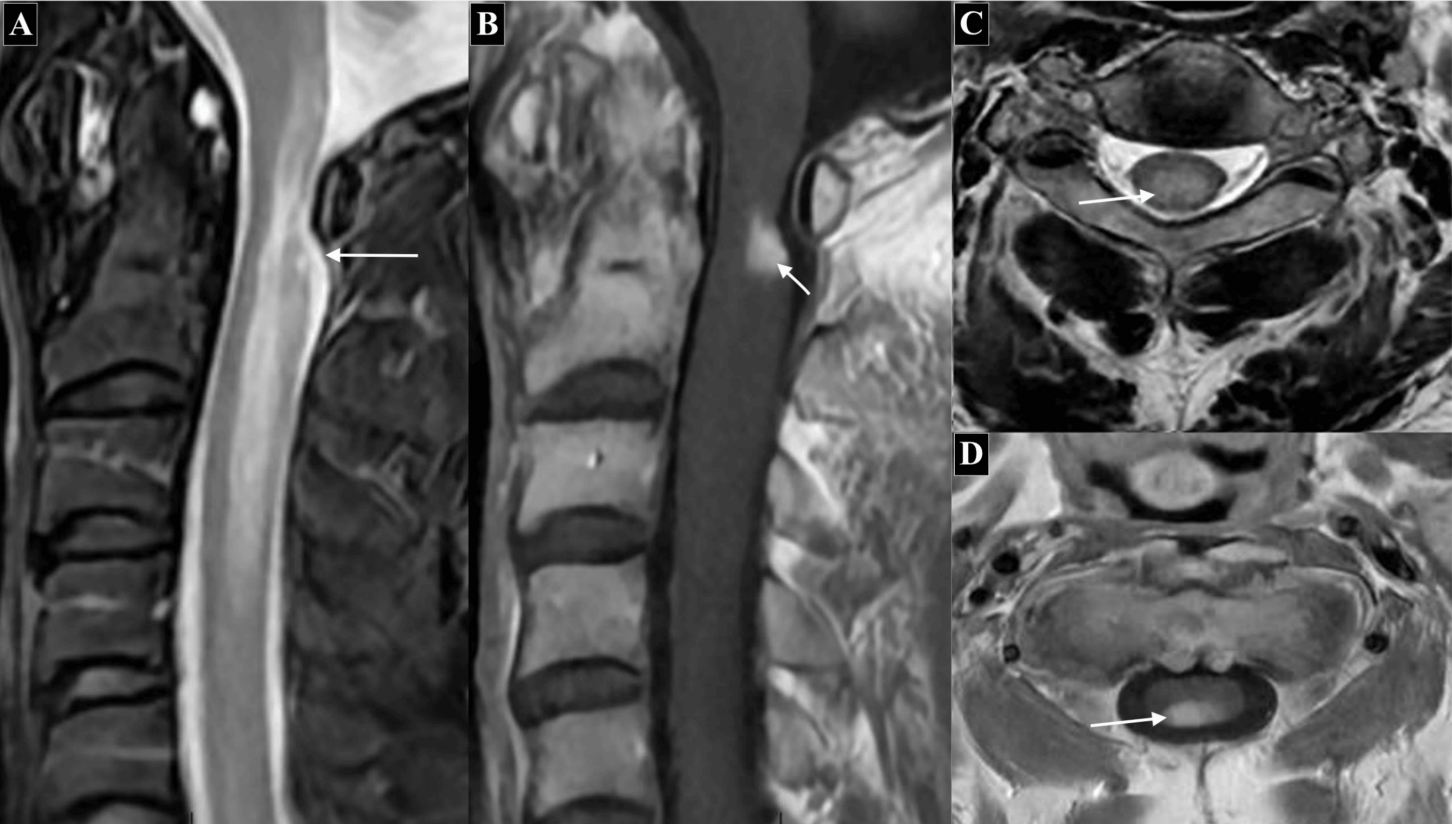
Preoperative T2 (A, C) and gadolinium-enhanced T1 (B, D) weighted axial MRI showed cervical T2 hyperintense lesion with focal nodular enhancement MRI: magnetic resonance imaging

Contrast-enhanced MRI identified a 6.4 mm intraspinal lesion with focal nodular enhancement, initially suggestive of myelitis (Figure 2).

**Figure 3:**
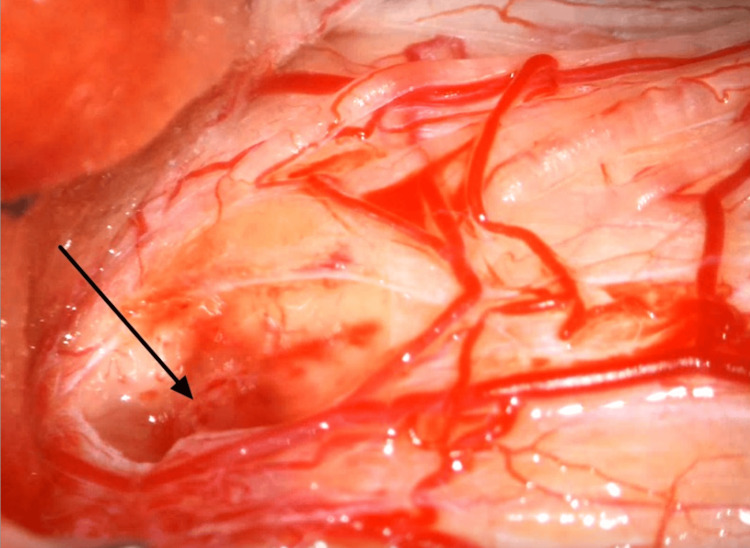
Intraoperative microscopic view of the lesion (arrow). The lesion appeared grayish, consistent with tumor tissue

**Figure 4:**
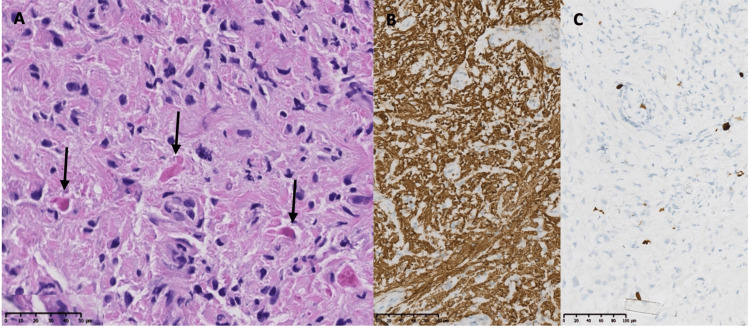
Histopathological and immunohistochemical findings. (A) H&E staining revealed a low-grade glial tumor with numerous Rosenthal fibers (black arrows). (B) The tumor cells were positive for GFAP. (C) The Ki-67 labeling index was observed in up to 3% of the tumor cells H&E: hematoxylin and eosin, GFAP: glial fibrillary acidic protein

Further investigation excluded an inflammatory etiology, and CSF analysis showed absence of oligoclonal band. The patient did not respond to steroid therapy. Differential diagnosis included an intramedullary tumor process such as hemangioblastoma or ependymoma, and following a multidisciplinary board, surgical exploration was decided. A C2 laminectomy with excision of the posterior arch of C1 was performed, followed by durotomy. Intraoperatively, the lesion appeared grayish, consistent with tumor tissue, but poorly delineated (Figure 3) and difficult to dissect from the normal spinal cord. Consequently, a near-complete excision was performed. Histopathological analysis confirmed the presence of a pilocytic astrocytoma (Figure 4).

Postoperative physical examination revealed a mild posterior cord syndrome with a favorable progression after physiotherapy. At the three-month postoperative follow-up, the patient no longer experienced cervicobrachialgia and showed a 70% improvement in paresthesia. The residual lesion was stable on MRI at six months.

## Discussion

PA in adults is notably rarer than in children, with limited case reports and series available. Typically, these lesions predominantly affect the cervical segment [4] and appear iso- or hypo-intense on T1-weighted images (WI) and hyperintense on T2-WI. MRI contrast enhancement can be homogeneous or patchy, but some tumors also show no enhancement.

Differentiating spinal PA from other intramedullary lesions such as ependymomas or hemangioblastomas remains a challenge. Syringomyelia is associated with spinal PA, with some series reporting a higher frequency of syringomyelia compared to other spinal astrocytomas [3].

Spinal PA rarely invades the surrounding tissue, usually maintaining a clear margin between the tumor and normal spinal cord parenchyma [3]. Gross total resection is possible in 50-81% of cases and is considered the gold standard, provided that neurological function preservation is maintained [5,6]. In this case, the poorly delineated borders of the tumor made complete resection challenging. A near-complete excision was performed cautiously to avoid neurological deficits, resulting in favorable outcomes. The use of intraoperative evoked potentials might have allowed for a more optimal and secure extent of resection. While gross total resection is considered the standard of care, complete resection is not always feasible due to the tumor's location and the risk of neurologic compromise.

In the literature, survival rates range between 20% [7] and 37.5% [8], despite high rates of gross total resection. Some studies suggest that the extent of resection does not significantly impact the recurrence rate [2], though this remains a topic of debate. Factors influencing recurrence include genetic mutations (e.g., NF-1 and H2-K27M) and the number of segments involved [8]. Recurrent cases underscore the need for vigilant follow-up.

The role of adjuvant therapies in spinal PA, such as radiotherapy or chemotherapy, remains unclear. Postoperative adjuvant radiotherapy for spinal PA is generally not recommended [9]. More comprehensive studies are required to better define the indications for adjuvant treatments in spinal PA. The role of steroids in patients with spinal PA is also unclear, and further research with larger patient cohorts is needed to clarify their efficacy [10].

## Conclusions

Spinal cord PA in adults is an exceedingly rare and diagnostically challenging condition. This case underscores the importance of considering PA in the differential diagnosis of spinal cord lesions, particularly when radiological features suggest myelitis or other intramedullary tumors. Despite its rarity, PA should remain a consideration due to its significant impact on patient outcomes when treated appropriately. Future research is needed to better understand the factors influencing recurrence, such as genetic mutations, and to clarify the role of adjuvant therapies in treatment strategies. Larger studies focusing on the adult population are particularly needed, as most available data currently stem from pediatric cases. This will help refine treatment protocols and improve long-term outcomes for adult patients with spinal cord PA.
